# The Impact of Isoniazid Resistance on the Treatment Outcomes of Smear Positive Re-Treatment Tuberculosis Patients in the State of Andhra Pradesh, India

**DOI:** 10.1371/journal.pone.0076189

**Published:** 2013-10-11

**Authors:** Dorai Deepa, Shanta Achanta, Jyoti Jaju, Koteswara Rao, Rani Samyukta, Mareli Claassens, Ajay M. V. Kumar, Vishnu PH

**Affiliations:** 1 State Tuberculosis Training and Demonstration Center, Directorate General of Health Services, Government of Andhra Pradesh, Hyderabad, India; 2 World Health Organization (WHO) Country Office in India, New Delhi, India; 3 State TB Cell, Directorate General of Health Services, Ministry of Health and Family Welfare, Government of Andhra Pradesh, Hyderabad, India; 4 Desmond Tutu Tuberculosis Centre, Department of Pediatrics and Child Health Stellenbosch University, Cape Town, South Africa; 5 International Union Against Tuberculosis and Lung Disease, South-East Asia Regional Office, New Delhi, India; Institut de Pharmacologie et de Biologie Structurale, France

## Abstract

**Background:**

Multi drug resistant and rifampicin resistant TB patients in India are treated with the World Health Organization (WHO) recommended standardized treatment regimens but no guidelines are available for the management of isoniazid (INH) resistant TB patients. There have been concerns that the standard eight-month retreatment regimen being used in India (2H_3_R_3_Z_3_E_3_S_3_/1H_3_R_3_Z_3_E_3_/5H_3_R_3_E_3_; H-Isoniazid; R-Rifampicin; Z-Pyrazinamide; E-Ethambutol; S-Streptomycin) may be inadequate to treat INH resistant TB cases and leads to poor treatment outcomes. We aimed to assess if INH resistance is associated with unfavorable treatment outcomes (death, default, failure and transferred out) among a cohort of smear positive retreatment TB patients registered in three districts of Andhra Pradesh, India.

**Methods:**

We conducted a retrospective record review of all smear positive retreatment TB patients without rifampicin resistance registered during April–December 2011.

**Results:**

Of 1,947 TB patients, 1,127 (58%) were tested with LPA—50 (4%) were rifampicin resistant, 933 (84%) were sensitive to INH and rifampicin and 144 (12%) were INH resistant. Of 144 INH resistant cases, 64 (44%) had poor treatment outcomes (25 (17%) default, 22 (15%) death, 12 (8%) failure and 5 (3%) transfer out) as compared to 287 (31%) among INH sensitive cases [aRR 1.46; 95%CI (1.19–1.78)].

**Conclusion:**

Our study confirms that INH resistance is independently associated with unfavorable treatment outcomes among smear positive retreatment TB patients, indicating that the current treatment regimen may be inadequate. These findings call for an urgent need for randomized controlled trials to discover the most effective treatment regimen for managing INH resistant TB.

## Introduction

Resistance to isoniazid (INH) is the most common form of mono resistance with a prevalence of 10% among new tuberculosis (TB) cases and 28% among retreatment cases reported in 2009 globally [1].While there is adequate consensus on the diagnosis and management of multidrug resistant tuberculosis (MDR-TB) patients at global and national levels, the guidance on the management of other types of resistance, including INH resistance, has been widely debated [2,3].WHO currently recommends for INH resistant TB patients, a daily regimen of Rifampicin, Pyrazinamide and Ethambutol for a period of 6-9 months with added fluoroquinolone among those with extensive disease[4]. However, the Revised National TB Control Programme (RNTCP) in India continues to use the standard eight-month retreatment regimen, administered thrice-weekly (2H_3_R_3_Z_3_E_3_S_3_/1H_3_R_3_Z_3_E_3_/5H_3_R_3_E_3_; H-Isoniazid; R-Rifampicin; Z-Pyrazinamide; E-Ethambutol; S-Streptomycin) for TB patients with INH resistance. Though there have been concerns that this regimen may be inadequate to treat the INH resistant TB cases and may lead to poor treatment outcomes, there is limited published information on this topic from India. The only study from India on this topic by Vijay et al in 1999-2001 reported a high proportion of loss to follow-up (~44%) among a cohort of smear positive retreatment TB patients, while failure rates were low and acquired rifampicin resistance was low at 1.8% [5]. Thus, they concluded that retreatment regimen was effective for all types of resistance other than MDR-TB, provided adherence was ensured while studies conducted elsewhere in the world [6–8] and a meta-analysis [9] conducted in 2009 reported poor outcomes among INH resistant TB. 

The RNTCP of India introduced the programmatic management of drug resistant TB services (PMDT) in 2007 and is now scaling up services across the country. As per PMDT guidelines [10], all retreatment smear positive TB cases at diagnosis are offered a Line Probe Assay (LPA), a test for assessing drug susceptibility to INH and rifampicin. Patients found to be MDR-TB and rifampicin mono-resistant are initiated on a 24 month standard MDR-TB treatment regimen[10]. However, those with INH resistance (without resistance to rifampicin) receive the eight-month retreatment regimen. In this operational research, we aimed to assess if INH resistance was associated with poor treatment outcomes among retreatment smear positive TB patients managed under routine program settings in India. The specific objectives were to assess among a cohort of retreatment smear positive TB patients registered in the RNTCP in Andhra Pradesh, South India, i) the number (proportion) tested for drug susceptibility using LPA at diagnosis ii) the number (proportion) diagnosed as having INH resistance and iii) treatment outcomes among those with and without INH resistance.

## Methods

### Ethics considerations

Since this was a retrospective record review, we could not obtain written informed consent from individual patients. The study protocol was reviewed and ethics clearance (including a waiver of informed consent) was provided by the Ethics Advisory Group of International Union Against Tuberculosis and Lung Disease (The Union) and the Ethics committee of the state of Andhra Pradesh. Local administrative approvals were obtained for the record review.

### Study design & setting

Retrospective cohort study involving review of records routinely maintained under RNTCP.

Andhra Pradesh is a state in the southern part of India with a population of 85 million. According to a Drug Resistance Surveillance study done in Andhra Pradesh, India during 2008-09, the prevalence of MDR-TB was 2.1% among new TB cases and 12% among the retreatment TB cases[10]. In the same study, the prevalence of any INH resistance among retreatment cases was around 28.6% (25.6-31.6). Of 24 districts in the state, three districts (Hyderabad, Rangareddy, Medak) with a population of approximately 12.6 million, were selected for the study. These districts were selected because they have been implementing PMDT since 2010 and had access to LPA[10]. As per PMDT guidelines, all retreatment smear positive TB cases should be offered drug susceptibility testing using LPA. Sputum samples from these cases are collected in peripheral health institutions and transported to a centrally located intermediate reference laboratory (IRL) which is accredited nationally to perform LPA. Patients with confirmed MDR-TB or rifampicin monoresistance receive standardized 24-month second line TB treatment (6-9 months of intensive phase with six drugs - pyrazinamide, ethambutol, ethionamide, cycloserine, levofloxacin and kanamycin and 18 months of continuation phase with four drugs - ethambutol, ethionamide, cycloserine and levofloxacin)[10]. The patients with INH resistance (without rifampicin resistance) receive the standardized retreatment regimen as mentioned above. All patients receive treatment under directly observed therapy (DOT).All case definitions and outcomes are standardized and in line with WHO guidelines [3].

### Study population & Sample size

We included all consecutive smear positive retreatment TB patients registered for treatment in three selected districts of Andhra Pradesh from April 2011 to March 2012.Patients registered as ‘transfer-in’ were excluded.

The sample size was calculated assuming a 15% difference in treatment success rates between INH resistant (exposed with assumed treatment success of 65%) and INH susceptible (unexposed with assumed treatment success of 80%) TB patients, 95% confidence, 80% power and a ratio of 1:3 between exposed to unexposed. 95 INH resistant TB cases and 285 sensitive cases were needed to achieve the sample size. Considering the number of patients whose sputum samples are tested per month with LPA in the study districts, it was decided to enroll all patients registered from April 2011 to March 2012.

### Data collection, validation, entry and analysis

We extracted information on the following variables – name of district, age, sex, TB number, type of previously treated TB case, HIV status and treatment outcomes - into a structured data collection format by reviewing the TB registers maintained by the senior treatment supervisors of the concerned districts. The information on whether these patients’ samples were tested with LPA and results of the tests were extracted from culture and drug susceptibility (DST) registers at the IRL.

Data were double entered, validated and analyzed using EpiData statistical software (Version 3.1 for entry, Version 2.2.2.178 for analysis, EpiData Association, Odense, Denmark). For the purpose of analysis, we categorized outcomes into two categories – favorable (cured and treatment completed) and unfavorable (death, loss-to-follow-up (referred to as ‘default’ in standard WHO nomenclature), failure, transferred out and switched to MDR-TB treatment). Univariate analysis was done and appropriate proportions were calculated to describe the demographic and clinical profile of TB patients. Bivariate analysis was done to examine possible associations of demographic and clinical variables with treatment outcomes. Relative Risks (RR) and 95% confidence intervals (CI) were calculated. Chi square or Fischer’s exact tests were used, as applicable, to compare proportions and a P-value of <0.05 was considered statistically significant. To assess the independent effects of each variable after adjusting for other variables, a multivariate analysis using log-binomial regression was done and adjusted relative risks were calculated using STATA (StataCorp. 2011. Stata Statistical Software: Release 12. College Station, TX: StataCorp LP.). All the factors found to be statistically significant during bivariate analysis were included in the multivariate analysis.

## Results

**Figure 1:**
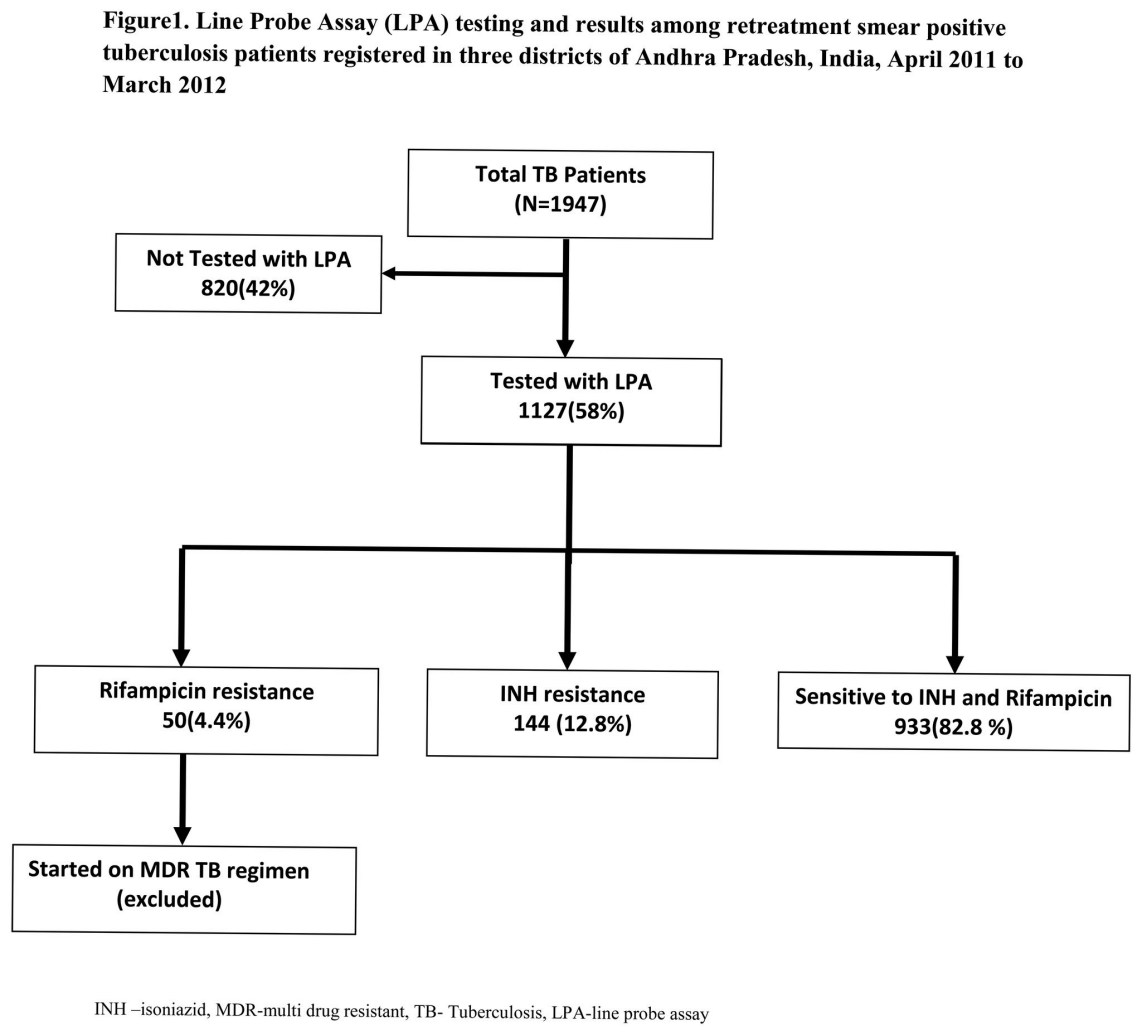
Line Probe Assay (LPA) testing and results among retreatment smear positive tuberculosis patients registered in three districts of Andhra Pradesh, India, April 2011 to March 2012.

The selection of study participants in described in Figure **1**.Of 1947 patients registered, 1127 (58%) were tested with LPA. Of 1127, 933 (84%) were sensitive to INH and rifampicin, 50 (4.4%) were rifampicin resistant and 144 (12%) were INH resistant. 

Demographic and clinical characteristics associated with LPA testing are shown in Table **1**. Patients registered as ‘failure’ and HIV positive patients were more likely to be tested with LPA.

Baseline demographic and clinical characteristics between those with and without INH resistance are compared in Table **2**. The two groups were similar with respect to all characteristics except sex; there was higher proportion of females in INH resistant group.

**Table 1:** Demographic characteristics of smear positive retreatment tuberculosis patients registered in three districts of Andhra Pradesh, India, by LPA testing status, April 2011 to March 2012.

**Characteristic**	**Tested with LPA**	**Not tested with LPA**	**P value**
	**N (%)**	**N (%)**	
**Total**	1127 (100.0)	820 (100.0)	
**Age groups**			
<40 years	662 (58.7)	456 (55.6)	0.168
>40 years	465 (41.3)	364 (44.4)	
**Sex**			
Male	817 (72.5)	623 (76.0)	0.083
Female	310 (27.5)	197 (24.0)	
**Type of retreatment case**			
Relapse	579 (51.4)	449 (54.8)	**0.001**
Failure	110 (9.8)	43 (5.2)	
TAD	438 (38.9)	328 (40.0)	
**HIV Status**			
Negative	1030 (91.4)	729 (88.9)	**<0.001**
Positive	70 (6.2)	43 (5.2)	
Unknown	27 (2.4)	48 (5.9)	

TAD-Treatment After Default; HIV-Human Immunodeficiency Virus; LPA-Line Probe Assay;

**Table 2:** Demographic characteristics of smear positive retreatment tuberculosis patients, by INH resistance registered in three districts of Andhra Pradesh, India April 2011 to March 2012 (N=1077)

**Characteristic**	**With INH resistance**	**Without INH resistance**	**P value**
	**N (%)**	**N (%)**	
**Total**	**144 (100.0)**	**933 (100.0)**	
**Age**			
<40 years	85 (59.0)	546 (58.6)	0.908
>40 years	59 (41.0)	387 (41.5)	
**Sex**			
Male	94 (65.3)	692 (74.2)	**0.002**
Female	50 (34.7)	241 (25.8)	
**Type**			
Relapse	72 (50.0)	483 (51.8)	0.667
Failure	17 (11.8)	88 (9.4)	
TAD	55 (38.2)	362 (38.8)	
**HIV Status**			
Negative	134 (93.1)	851 (91.2)	0.434
Positive	6 (4.2)	63 (6.8)	
Unknown	4 (2.8)	19 (2.0)	

INH-isoniazid; HIV-Human immunodeficiency virus; TAD-treatment after default

Treatment outcomes among patients with and without INH resistance are compared in Table **3**. Overall, 728(66%) had successful outcomes. The treatment success was worse among INH resistant patients (56%) as compared to INH sensitive (69%) patients due to higher death and default rates. Among patients who completed treatment (after excluding deaths, defaults and transfer outs), the failure rates were higher among INH resistant patients (13%) as compared to INH sensitive patients (10.5%).

**Table 3:** Treatment outcomes among smear positive retreatment tuberculosis patients, by isoniazid (INH) resistance, registered in three districts of Andhra Pradesh, India, April 2011 to March 2012

**Category**	**INH resistance**	**Without INH resistance**	**Total**
	**N (%)**	**N (%)**	**N (%)**
**Total**	144 (100.0)	933 (100.0)	1077 (100.0)
Cured	78 (54.2)	632 (67.7)	710 (65.9)
Treatment completed	2 (1.4)	14 (1.5)	16 (1.5)
Died	22 (15.3)	83 (8.9)	95 (8.8)
Failure	12 (8.3)	76 (8.1)	88 (8.2)
Default	25 (17.4)	115 (12.3)	140 (13.0)
Transferred out	5 (3.5)	12(1.3)	17 (1.6)
Not recorded	0 (0)	1(0.1)	1 (0.1)

Factors associated with unfavorable treatment outcomes are shown in Table **4**. On unadjusted analysis, INH resistance, male sex, those registered as ‘failure’ and unknown HIV status were found to be associated with unfavorable treatment outcomes. These associations persisted even after adjusting for each other during multivariate analysis. INH resistance was found to be independently associated with unfavorable treatment outcomes.

**Table 4:** Factors associated with unfavorable treatment outcomes among smear positive retreatment tuberculosis patients registered in three districts of Andhra Pradesh, India, April 2011 to March 2012 (N=1077)

**Characteristic**	**Unfavorable outcomes**	**Favorable outcomes**	**Unadjusted RR (95% CI)**	**Adjusted RR (95% CI)**
	**N (%)**	**N (%)**		
**Total**	351(32.6)	726 (67.4)		
**INH resistance**				
Yes	64 (44.4)	80 (55.6)	**1.44 (1.18-1.78)**	**1.46(1.19-1.78)**
No	287 (30.8)	646 (69.2)	Reference	Reference
Sex				
Male	276 (35.1)	510 (64.9)	**1.36 (1.10-1.69)**	**1.36(1.09-1.68)**
Female	75 (25.8)	216 (74.2)	Reference	Reference
**Age**				
<40 years	196 (31.1)	435 (68.9)	Reference	
>40 years	155 (34.8)	291 (65.2)	1.12(0.94-1.33)	
**Type**				
Relapse	158 (28.5)	397 (71.5)	Reference	Reference
TAD	142 (34.1)	275 (65.9)	1.20 (0.99-1.44)	1.18(0.98-1.42)
Failure	51 (48.6)	54 (51.4)	**1.71(1.35-2.16)**	**1.62(1.28-2.04)**
**HIV status**				
Negative	310 (31.5)	675 (68.5)	Reference	Reference
Positive	29 (42.0)	40 (58.0)	1.34 (1.00-1.79)	1.34 (1.00-1.77)
Unknown	12 (52.2)	11 (47.8)	1.66 (1.11-2.48)	1.68 (1.13-2.51)
**ART**				
Received	22 (36.2)	37 (63.8)	Reference	
Not received	7 (70.0)	3 (30.0)	1.93 (1.14-3.29)^#^	

ART- anti retroviral therapy; TAD-treatment after default; HIV- Human immunodeficiency virus; INH-isoniazid; RR-Relative Risk; CI-Confidence Interval Variables with RR and 95% CI in bold are statistically significant (P value < 0.05)

Unfavorable outcomes-died, default, transferred out and failure

Favorable outcomes-cured and treatment completed

# Fischer exact test

## Discussion

This is the largest cohort studied on this issue from India and confirms our hypothesis that INH resistance is independently associated with poor outcomes among smear positive retreatment TB patients. Patients with INH resistance had a 40% higher chance of unfavorable treatment outcomes mainly related to high default, death and failure rates. This is in concurrence with the findings of studies published worldwide [6–8]and that of the meta-analysis[9] published in 2009, thus adding to the evidence that the RNTCP recommended retreatment regimen is ineffective in treating INH resistant TB cases. The meta-analysis also showed that among INH resistant TB patients, the risk of relapse and acquired rifampicin resistance (ARR) was high. The risk of ARR was higher among patients treated with intermittent regimens as compared to those with daily regimens. A study from Chennai among ART-naïve, HIV-infected patients receiving thrice weekly intermittent regimens showed a high risk of ARR, among those with pre-treatment INH resistance [11]. This needs to be noted since thrice weekly intermittent regimens continue to be used in India. We could not report on both relapse and ARR in our study, since these are not routinely measured in programme settings. The most important finding of the meta-analysis was the lack of evidence in support of the standardized retreatment regimen[9]. With no evidence from randomized controlled trials (RCTs), this is an area in need of urgent research. While awaiting the results of RCTs, a change the RNTCP can consider is to transition from using intermittent regimens to daily regimens as per WHO recommendations[2].

In addition to INH resistance, treatment outcomes were worse among males, those registered as ‘failure’ and people with unknown HIV status indicating sub-groups that need additional attention. Male patients in our study experienced worse outcomes similar to other studies [12]where it was attributed to smoking, alcoholism and liver problems, although we did not investigate the reasons. Patients with an unknown HIV status had worse outcomes than those with a known status. We speculate that this could be due to undiagnosed HIV and reinforces the need for universal HIV testing among TB patients.

There were a number of findings of programmatic importance. First, only 58% of all retreatment cases underwent LPA testing despite the recommendation that all should be tested. This is a cause of concern and the reasons have to be investigated. We found that “failure” cases and HIV positive cases were more likely to be tested with LPA as compared to others. This probably indicates persisting provider beliefs of selective referral of only those at highest risk, a previous recommendation under RNTCP. Health care providers need to be adequately sensitized about the new guidelines and need for offering LPA routinely to all retreatment smear positive TB patients. The other possible reason for the low proportion tested could be related to the challenges in arranging sputum collection and transport from the peripheral facilities to a central laboratory located in the capital city of the state. This partially mitigates the advantages of a rapid molecular method like LPA with a short turn-around time. Given the challenge of decentralizing availability of LPA due to infrastructural, human resource and training requirements, other molecular technologies including automated nucleic acid amplification tests should be considered. Second, the proportion of rifampicin resistance among smear positive retreatment patients in our cohort was 4.4% which is rather low as compared to 12% found in the drug resistance surveillance in the state. Also, the proportion of HIV positive patients was also relatively low at ~5% compared to about 10% observed in new TB patients[13]. We do not know the reasons for this difference, but it needs further investigation. It could be possible that most MDR-TB cases, especially among HIV-infected individuals, were undiagnosed and died before starting treatment – the reason for the apparent low proportion of MDR-TB and HIV positive patients in our treatment cohorts. A previous study from the state had shown substantial drop-outs from the identification of a presumptive MDR-TB patient to diagnosis and treatment[14]. This needs to be noted and warrants detailed investigation into the programmatic processes, from identification of MDR-TB suspect to diagnosis of MDR-TB and treatment initiation. Third, nearly one in five patients were lost to follow-up during treatment. This is unacceptable and calls for immediate attention by the programme managers. Reasons have to be investigated and appropriate corrective measures should be initiated. A previous study assessing the reasons for high default had shown that patients who received treatment from community based DOT providers were less likely to default than those receiving from health institution based care providers[12].This strategy could be implemented on a larger scale. 

### Limitations

First, our study was limited by its retrospective design and the operational nature of the study relying on accuracy of records routinely maintained by the programme. However, we feel that the supervision and monitoring system is robust and likely to have minimal impact on the results. Second, we had no information on the number of previous treatment episodes each patient had, which is one of the important confounders for treatment outcome. This might have influenced the treatment outcomes. Third, as the testing was done with LPA, susceptibility of these patients to other drugs (such as streptomycin and ethambutol) was not known. Patients with INH resistance could have had additional resistance to streptomycin and/or ethambutol which might have influenced the treatment outcomes. Fourth, while HIV positive patients had a higher risk of poor treatment outcome, especially if they did not receive ART, the numbers were too small to achieve statistical significance.

## Conclusion

INH resistance was independently associated with unfavorable treatment outcomes among smear positive retreatment TB patients. There is a need for intensive monitoring of patients with INH resistance. RCTs are urgently required to discover the best way of managing INH resistance. Meanwhile, the RNTCP should consider transitioning from using an intermittent treatment regimen to daily dosing for TB patients in India.
